# Risk factors and prevalence of toxocariasis in pregnant women and diabetic patients compared to healthy adults in Ilam province, western Iran

**DOI:** 10.17179/excli2018-1630

**Published:** 2018-10-23

**Authors:** Vahid Raissi, Zahra Sohrabi, Muhammad Getso, Omid Raiesi, Saeideh Hashemi Hafshejani, Hajar Shabandoust, Soudabeh Etemadi

**Affiliations:** 1 Department of Medical Parasitology and Mycology, School of Public Health, Tehran University of Medical Sciences, Tehran, Iran; 2 Student Research Committee, School of Nursing and Midwifery, Isfahan University of Medical Sciences, Isfahan, Iran; 3 Department of Parasitology and Mycology, School of Medicine, Kerman University of Medical Sciences, Kerman, Iran

**Keywords:** toxocariasis, pregnancy, diabetic patients, Ilam, Iran

## Abstract

Toxocara is one of the common intestinal nematodes in dogs and cats and is the agent of tissue migratory larvae in humans. Customarily, the prevalence of human toxocariasis hovers around 15.8 % in Iran. Furthermore, other research outcomes demonstrated a tendency for an outbreak of toxocariasis in Iran. Therefore, we carried out a cross-sectional study and assessed the seroprevalence of toxocariasis humans in Ilam Province, western of Iran. A total of 539 serum samples were collected between September 2017 and March 2018 from patients referred to the Health Centers of Ilam province, Iran. Serum samples were investigated for the presence of Toxocara using IgG antibodies, ELISA (Enzyme-Linked Immunosorbent Assay) kit. Risk factors such as contact with cats and dogs, living in rural areas were investigated among the study population. Out of 539 total samples collected, 97 cases (17.99 %) were positive for anti-toxocara IgG antibodies. These antibodies were recovered from serum samples of otherwise healthy adults (15.54 %, 49/296), pregnant women (21.16 %, 40/189) and diabetic patients (14.81 %, 8/54). This study showed significant relationship between toxocariasis and contact with animal pets in all studied groups (P value ≤ 0.05) and a significant relationship between toxocariasis and living in rural areas among pregnant women (P value ≤ 0.05).

## Introduction

Toxocariasis is a parasitic infection caused by *Toxocara canis* and *Toxocara cati,* mainly due to ingestion of the parasite eggs containing the third stage larva. Millions of people overall are exposed to toxocariasis, and several studies have displayed that toxocariasis has effects on human health. Highest prevalence of toxocariasis is seen in South-East Asia. Reports show that Toxocara larvae, can penetrate into the narrow intestinal mucosa and migrate to the liver, lungs, skeletal muscles, the brain, the eye, and other parts of the body, leading to a visceral migratory larval syndrome in humans (Negri et al., 2013[18]). Human is mainly afflicted with 3 major forms of toxocariasis, including Visceral Larva Migrans, Covert Toxocariasis and Common Toxocariasis. Covert (hidden, latent or occult) toxocariasis is a form of disease that can be seen in people with positive anti-toxocariasis antibodies. These patients have no specific clinical symptoms, and eosinophilia is not seen in all the patients (Momeni et al., 2016[17]; Ma et al., 2018[15]). Identification of the parasite is by direct observation of larva through biopsy, serologic and molecular methods. In most cases, albendazole is used to treat and remove larvae when the eye and brain are involved (Negri et al., 2013[18]). The occurrence in soil, human and animal, of toxocariasis in Iran has been reported to about 21.6 % on average. The prevalence of human toxocariasis cases in Iran was 15.8 % and the occurrence of Toxocara eggs in the soil sample was 26.8 % (Abdi et al., 2012[1]). The seroepidemiology study of toxocariasis has been reported in pregnant women worldwide, including Brazil (4.6 % of 280 instances) and China (8.62 % of 545 instances) which was mainly due to the interaction of pregnant women with soil polluted with dog and cat feces (Lescano et al., 1998[13]; Roldán et al., 2009[21]). In rare cases, a positive toxocariasis was reported in persons with diabetes who happened to be infected by a person with an untreated liver toxocariasis (Park et al., 2002[19]). The aim of this study was to determine the occurrence of toxocariasis in different groups of subjects and also to determine the risk factors in the individuals and compare them with each other (based on age, sex, place of living and association with the animals infected with parasites).

## Materials and Methods

A cross-sectional study was conducted in Ilam province, Iran. People who participated in as the study were clinically healthy individuals, pregnant women, and diabetic patients. A total of 539 participants were recruited between 23 September 2017 and 16 March 2018, comprised of 296 clinically healthy individuals, 189 pregnant women, and 54 diabetic patients. A structured questionnaire was used for collecting bio data and for assessing risk factors such as age, sex, area of residence; history of contact with dogs and cats and exposure to contaminated soil we reset. Approximately 5 ml of venous blood samples were drawn from each participant. Blood samples were left overnight at room temperature to allow clotting and centrifuged at 2000 RPM for 10 min. The serum was collected in Eppendorf tubes and stored at 4 °C for not more than 24-72 h and transported in an ice box to Laboratory of Parasitology, School of Public Health, Tehran University of Medical Sciences, Tehran Province where they were kept at −20 °C until tested. Serum samples were detected for anti-Toxocara IgG antibodies using an Enzyme-Linked Immunosorbent Assay “Toxocara” kit (IBL International GmbH, Hamburg, Germany). Absorbance reading equal to or greater than 0.38 OD units were considered to be positive. All tests were performed following the instructions of the manufacturer. The strength of association between dependent (IgG seropositivity to Toxocara) and independent variables: age, sex, location area, contact with dogs and cats and exposure with soil, was inferred by univariate logistic regression analysis using the SPSS 20 software package. Both dependent and independent variables were dichotomous variables. Odds ratio (OR) values were considered statistically significant within the 95 % CI. And Probability (P) value ≤ 0.05 was considered as statistically significant in all the analyses.

## Results

### Seroprevalence among clinically healthy individuals

Healthy adults were randomly selected from those referred for health screenings in Ilam province. Table 1(Tab. 1) shows the relationship between ages, sex, contact with dogs and cats, and place of residence of people with toxocariasis. A total of 270 persons aged 22-75 years (average 38.9 years) participated in this study. Most healthy adults were between the ages of 31-40 (46.67 %). Forty-nine cases of healthy people (15.54 %) were seropositive for anti-Toxocara antibodies (IgG). Often healthy adults with toxocariasis were men and most cases of toxocariasis in this group reside in villages and in close relation with dogs and cats. Majority of the seroprevalence of Toxocara infection was detected in clinically healthy individuals aged ≥ 40 years old (56.67 %). In addition, 95.9 % of these healthy adults have history of contact with dogs and cats (Table 1(Tab. 1)). Analysis of serum of healthy adults showed that there was no significant difference between age, sex and place of residence of people with seropositive cases of toxocariasis, but the association between dogs and cats has been a risk factor for toxocariasis.

### Seroprevalence among pregnant women

A total of 189 pregnant women who sought to healthcare at Ilam province were screened for anti-Toxocara IgG antibodies. The mean age of the 189 pregnant women who participated in the study was 31.2 years (range 18-41), among which 21.16 % were positive for anti-toxocariasis antibodies. Approximately 97.5 % (39/40) of the seropositive cases have history of interaction with cats and dogs. Most (92.5 %) of the seropositive pregnant women were rural residents. In pregnant women, there was a significant relationship between place of residence and association with dogs and cats among toxocariasis seropositive women (Table 2(Tab. 2)).

### Seroprevalence among diabetic patients

Fifty-four diabetic patients participated in this study among which 14.81 % were seropositive for Toxocara antibodies. Their ages ranged between 35 to 61 years old. All of the cases of toxocariasis had contact with dogs and cats. There was statistically significant difference between contact with cats and dogs and seropositivity among diabetic patients (Table 3(Tab. 3)). 

## Discussion

In this study we investigated seroprevalence of toxocariasis among healthy individuals, pregnant women, and diabetic patients of Ilam province in western Iran. Prevalence of toxocariasis among different population groups has been reported by many researchers. A study by Negri et al. (2013[18]) was conducted at the Center for Hematology, in South eastern Sao Paulo, Brazil 7.8 % of the 253 blood donors reported positive for anti-toxocariasis IgG antibody, while contact with contaminated soil was reported as the major risk factor for toxocariasis among the study group but not age nor gender. In the present study, we reported a seroprevalence of anti-toxocariasis IgG antibody (17.99 %) higher than that reported in Brazil while contact with infected cats and dogs was identified as the major risk factors for toxocariasis but age and gender had no effect (similar to the study done in Brazil). In another study by Lescano et al. (1998[13]) conducted in Lima Hospital in Peru reported, 33.7 % of the patients were positive for anti-toxocariasis IgG-antibody mostly among those exposed to contaminated soil. Similarly, the study in Peru also reported no significant differences in age and sex among the studied population. However, a different study in Peru demonstrated that male were mostly affected by toxocariasis, and the reason for this was the increased association of men with dogs and cats due to their occupation or social behaviours (Roldán et al., 2009[21]). Studies have been conducted in different parts of the world where lower prevalence of toxocariasis among healthy adults was reported than in the present study (Park et al., 2002[19]; De Savigny et al., 1979[6]). A study on the prevalence of toxocariasis in China was conducted on groups at risk for toxocariasis, which played an important role in developing the main idea of this study. Among three groups: healthy adults, pregnant women and patient with psychosocial problems and they reported 13.7 % and 9.19 % seroprevalence of anti-toxocariasis IgG antibodies among healthy adults and pregnant women, respectively. Although the current study reported higher prevalence of toxocariasis among pregnant women than healthy adults. However, similar results were observed in relation to the association between toxocariasis and contact with dogs and cats (Cong et al., 2014[5]). Compared to our findings, lower seroprevalence of toxocariasis among pregnant women were reported from Brazil. Santos et al. (2015[22]) reported toxocariasis seroprevalence of 4.6 % among 280 pregnant women in urban areas of Brazil while Pereira et al. (2016[20]) reported a higher prevalence of 7.4 % among 311 pregnant women. Similarly compared to our findings, both studies reported significant association between higher seroprevalence and history of contact with pets (Mizgajska, 2001[16]; Deutz et al., 2005[7]). Significant associations between seropositivity among individuals with history of contact with contaminated soil and dogs infected with toxocariasis have been reported in this study and many other studies from around globe (Mizgajska, 2001[16]; Deutz et al., 2005[7]; Berenji et al., 2016[2]; Wolfe and Wright 200[23]3; Colli et al., 2010[4]). Also, living in rural areas was reported as a risk factor for acquisition of toxocariasis in this as well as other previous studies (Gawor et al., 2008[8]; Habluetzel et al., 2003[9]). In the present study, close contact with infected animal pets (dogs and cats) was significantly associated with positive toxocariasis in diabetic patients while liver and pancreatic disorders were reported among diabetic patients infected with larval (Choi et al., 2012[3]; Lee et al., 1976[12]). Although not sought for in the current study, but recent evidences revealed that toxocariasis with CNS involvement has significant neuropsychological impact on its victims (Luna et al., 2018[14]).

## Conclusion

The overall seroprevalence of toxocariasis among apparently healthy adults, diabetic patients and pregnant women was estimated to be approximately 18 % in Ilam, Iran. To the best of our knowledge this is the first time, the prevalence of toxocariasis in diabetic patients in Iran was reported. The high seroprevalence of toxocariasis recorded in the current study shall be a matter of concern, especially among pregnant women. Toxocariasis is associated with animal pets and living in rural areas of the region. Further studies involving comprehensive neuropsychological evaluation of seropositive patients can be an important milestone in diagnosing neurotoxocariasis (Janecek et al., 2017[10]; Lawton and Sharma, 2017[11]; Luna et al., 2018[14]).

## Acknowledgements

Sincere gratitude of all professors and students of parasitology at the School of Public Health in Tehran University of Medical Sciences.

## Conflict of interest

The authors declare no conflict of interest.

## Figures and Tables

**Table 1:**
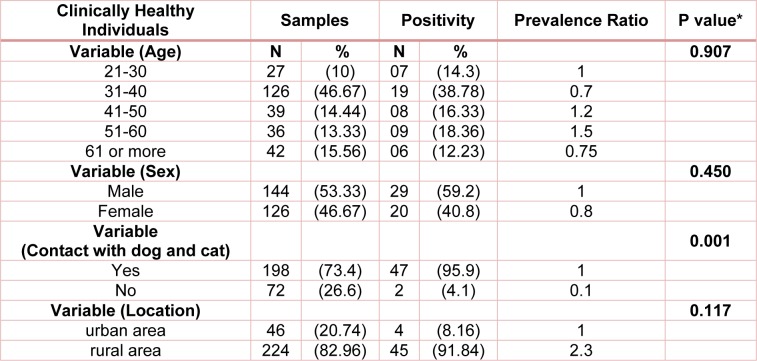
Seroprevalence among clinically healthy individuals referred for health screenings in Ilam province

**Table 2:**
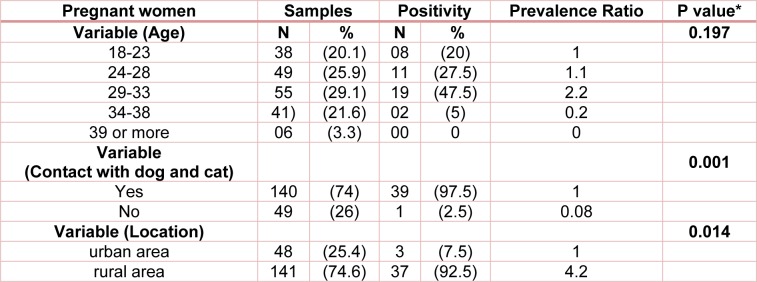
Toxocariasis seroprevalence among pregnant women screened at hospital of Ilam province

**Table 3:**
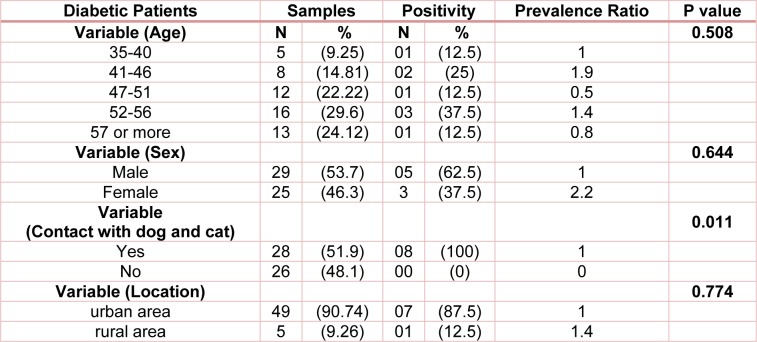
Seroprevalence of toxocariasis among diabetic patients of Ilam province, Iran
